# Neuronal representation of audio-place associations in the medial prefrontal cortex of rats

**DOI:** 10.1186/s13041-015-0147-5

**Published:** 2015-09-22

**Authors:** Qi Wang, Sheng-Tao Yang, Bao-Ming Li

**Affiliations:** Institute of Neurobiology & State Key Laboratory of Medical Neurobiology, Fudan University, Shanghai, 200032 China; Center for Neuropsychiatric Diseases, Institute of Life Science, Nanchang University, Nanchang, 330031 China

**Keywords:** Neuronal activity, Medial prefrontal cortex, Audio-place association, Rats

## Abstract

Stimulus-place associative task requires humans or animals to associate or map different stimuli with different locations. It is know that the medial prefrontal cortex (mPFC) in rats, also termed prelimbic cortex (PrL), is important for performing stimulus-place associations. However, little is known about how mPFC neurons encode stimulus-palce associations. To address this, the present study trained rats on an audio-place associative task, whereby the animals were required to associate two different tones with entering two different arms in a Y-shaped maze. Reversible inactivation of the mPFC by local infusion of the GABA_A_ receptor agonist muscimol severely impaired the performance of rats on the associative task, again indicating an important role of mPFC in the task performance. Single-unit recording showed that a group of mPFC neurons (40/275, 14.5 %) fired preferentially for the audio-place associations, providing the first electrophysiological evidence for the involvement of mPFC cells in representing audio-place associations.

## Introduction

The most significant difference in the prefrontal cortex (PFC) between rodents and primates is that, while the dorsolateral PFC (dlPFC) in monkeys has obvious granular layer (known as layer IV), the medial PFC (mPFC) in rodents, also termed prelimbic cortex (PrL), has no obvious granular layer. Despite of such difference, it has been documented that, the rodent mPFC is a cortical area similar to the monkey dlPFC in terms of connections and functions [1–3].

It is known that the dlPFC in monkeys plays a vital role in associative tasks [4–7]. For example, PFC lesions or inactivation disrupted the establishment of arbitrary associations between visual stimuli and behavioral choices [4, 6]. Electrophysiological studies showed that dlPFC neurons are involved in representing the response category of instruction cue in an associative task [8], and encoding the specific associations between sensory stimuli and behavioral choices [5, 7].

Stimulus-place associative task requires individuals to associate different stimuli with different spatial locations. For example, if stimulus A is presented, reward is conditional on selecting location A, and if stimulus B is presented, reward is conditional on selecting location B. Previous studies showed that the mPFC in rats is important for the task performance of stimulus-place associations [9]. However, it is unknown whether and how mPFC cells represent stimulus-place associations.

**Fig. 1:**
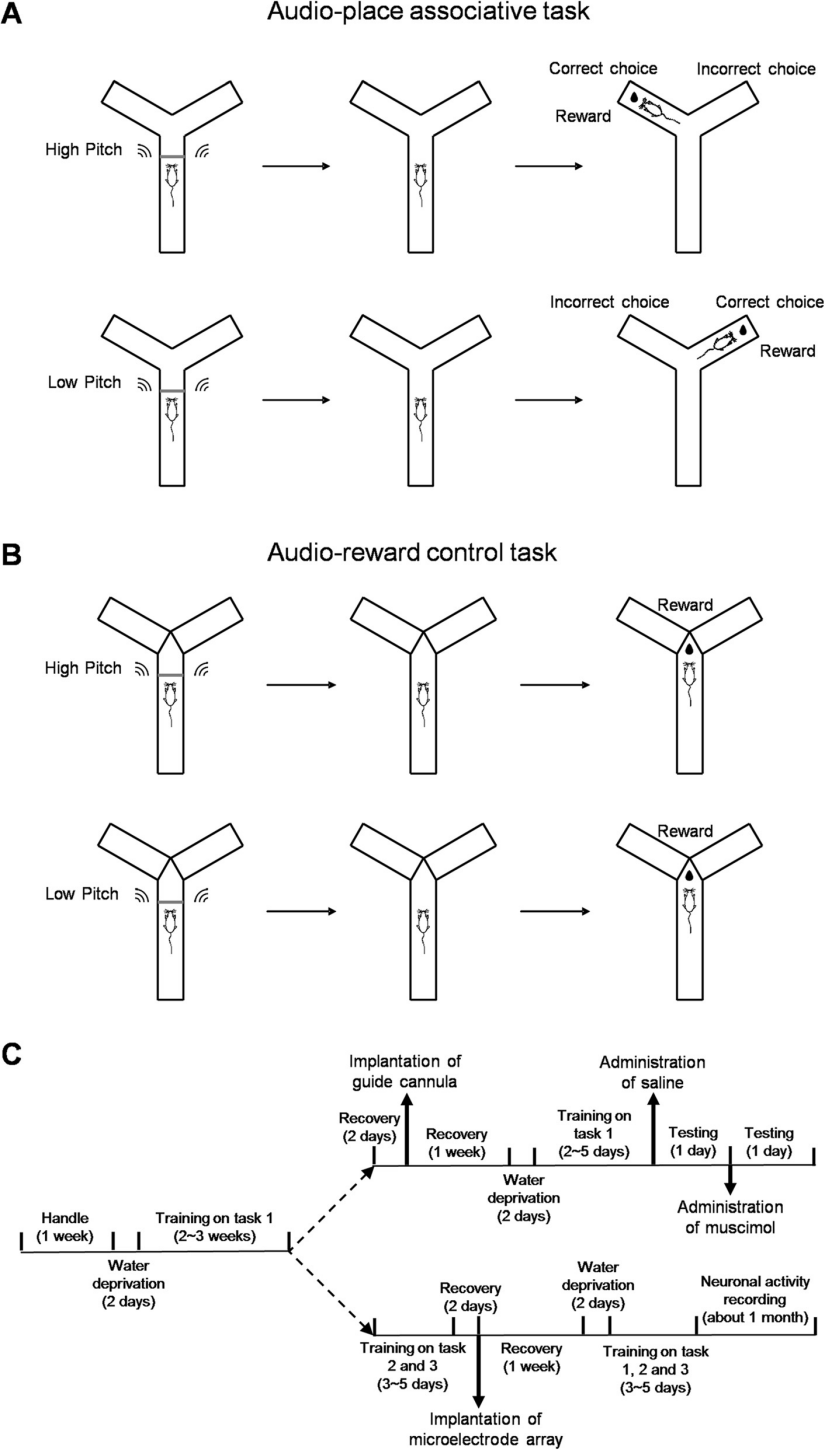
Schematic diagrams of the audio-place associative task (**a**), the audio-reward control task (**b**) and the timeline of experimental procedure (**c**). In the *audio-place associative task*, rats were placed in the start box of the Y-maze and an auditory cue (high- or low-pitch tone) was presented. The door was then withdrawn and rats were allowed to visit the left or right arm of the maze. Rats were required to visit the left arm if the cue was a high-pitch tone, or visit the right arm if the cue was a low-pitch tone. After a correct choice, rats were given water reward, which was delivered at the terminal of the visited arm. In the *audio-reward control task*, rats were placed in the start box of the maze, and the high- or low-pitch tone was presented, as in the *audio-place associative task*. Thereafter, the door was withdrawn, and rats were allowed to approach the water reward delivered at the intersection of the Y-maze. The whole experiments contained behavioral and electrophysiological parts. Once rats had learned the audio-place associative task (Task 1), some rats was used for behavioral experiments, whereby rats were implanted with guide cannula, and received intra-mPFC infusion of muscimol (or saline as control) to test the importance of mPFC for performing Task 1. Other rats were trained further on the arbitrary spatial choice task (Task 2), and subsequently on the audio-reward control task (Task 3). Thereafter, the rats were implanted with microelectrode arrays in the mPFC, and unit activities were recorded when the rats were performing the three tasks

In the present study, we trained rats on an audio-place associative task (Fig. 1a). In this task, rats were required to associate two different tones with two different arms in a Y-shaped maze. We found that mPFC cells demonstrated preferential firing for the audio-place associations.

## Results

### mPFC inactivation impairs the task performance

**Fig. 2:**
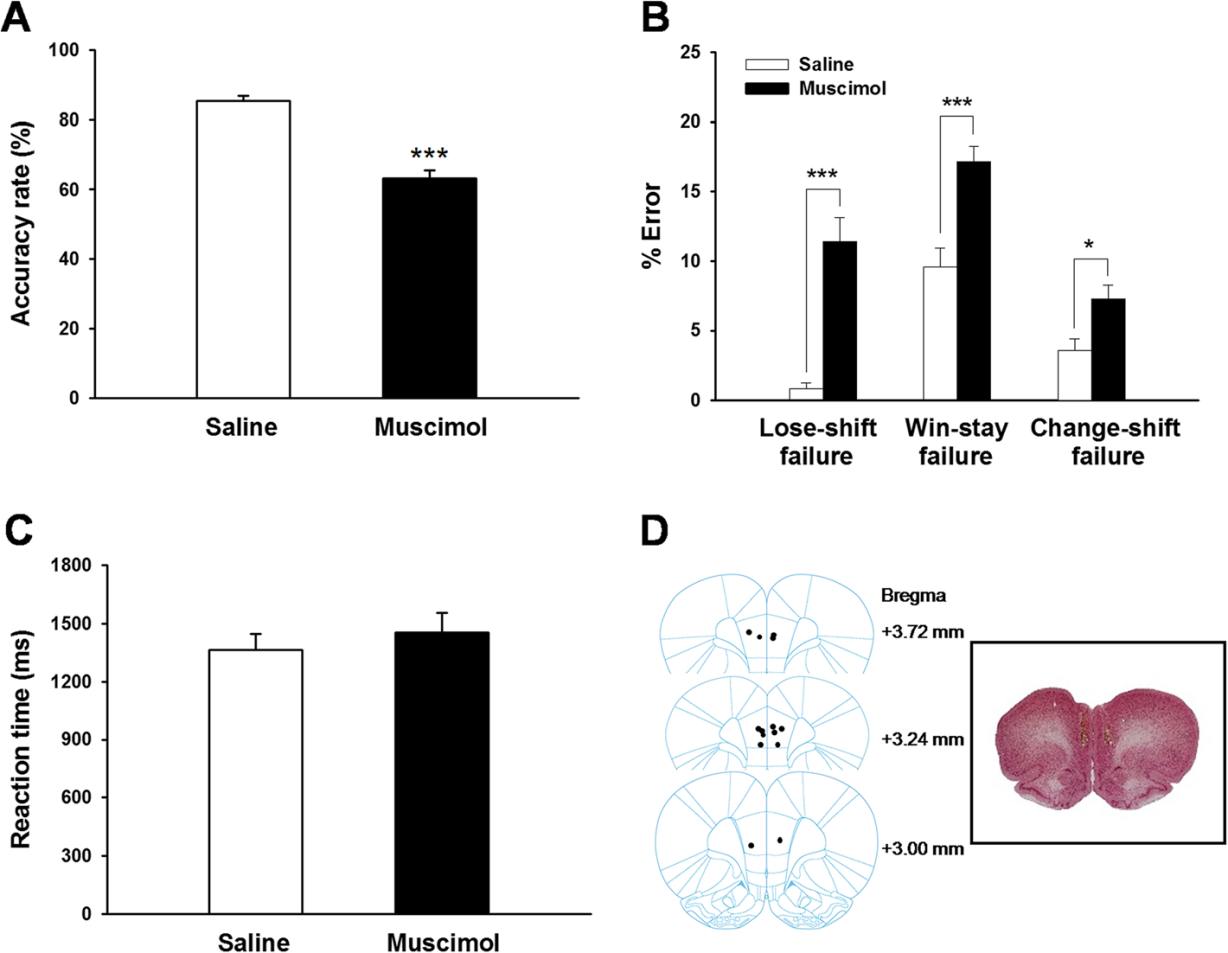
Intra-mPFC infusion of muscimol impaired the performance of rats on the audio-place associative task. **a** The correct rate of task performance was significantly reduced upon musicmol infusion. **b** The performance errors were expressed as a significant increase in lose-shift, win-stay and change-shift failures. **c** The reaction time, that is, the duration for rats to leave the start-box to enter the side arms, was not affected following musicmol infusion. **d** Histological reconstruction of the infusion sites in the mPFC. Bilateral infusions were performed, with 0.5 μg/0.5 μL (8.8 mmol/L) of muscimol each side, or equal volume of saline. *Insert*: a brain section showing an infusion trace. *n* = 7 rats for infusion. * *P* < 0.05, *** *P* < 0.001

In order to examine if the mPFC is necessary for the audio-place associative task performance, we locally infused the GABAergic agonist muscimol into the mPFC. Saline was infused as control. Histological examination with neutral red staining confirmed the locations of infusion in the mPFC (Fig. 2a). The correct rate of performance was significantly reduced upon intra-mPFC infusion of muscimol (Fig. 2b; *p* < 0.001 for muscimol vs. saline, paired *t*-test; *n* = 7 rats). Analysis of error types revealed that, the rats made significantly more lose-shift, win-stay, and change-shift failures after muscimol infusion (Fig. 2c). The animals demonstrated a comparable motor capability or response speed after muscimol infusion, as the reaction time was not changed (Fig. 2d; *p* = 0.35 for muscimol vs. saline, paired *t*-test).

### mPFC neurons encode audio-place associations

In order to explore the neuronal correlates of audio-place associations in the mPFC, single unit activities were recorded when rats performed the associative task. The arbitrary spatial choice task and audio-reward control task were introduced to help identify the functional significance of task-related activity.

**Fig. 3:**
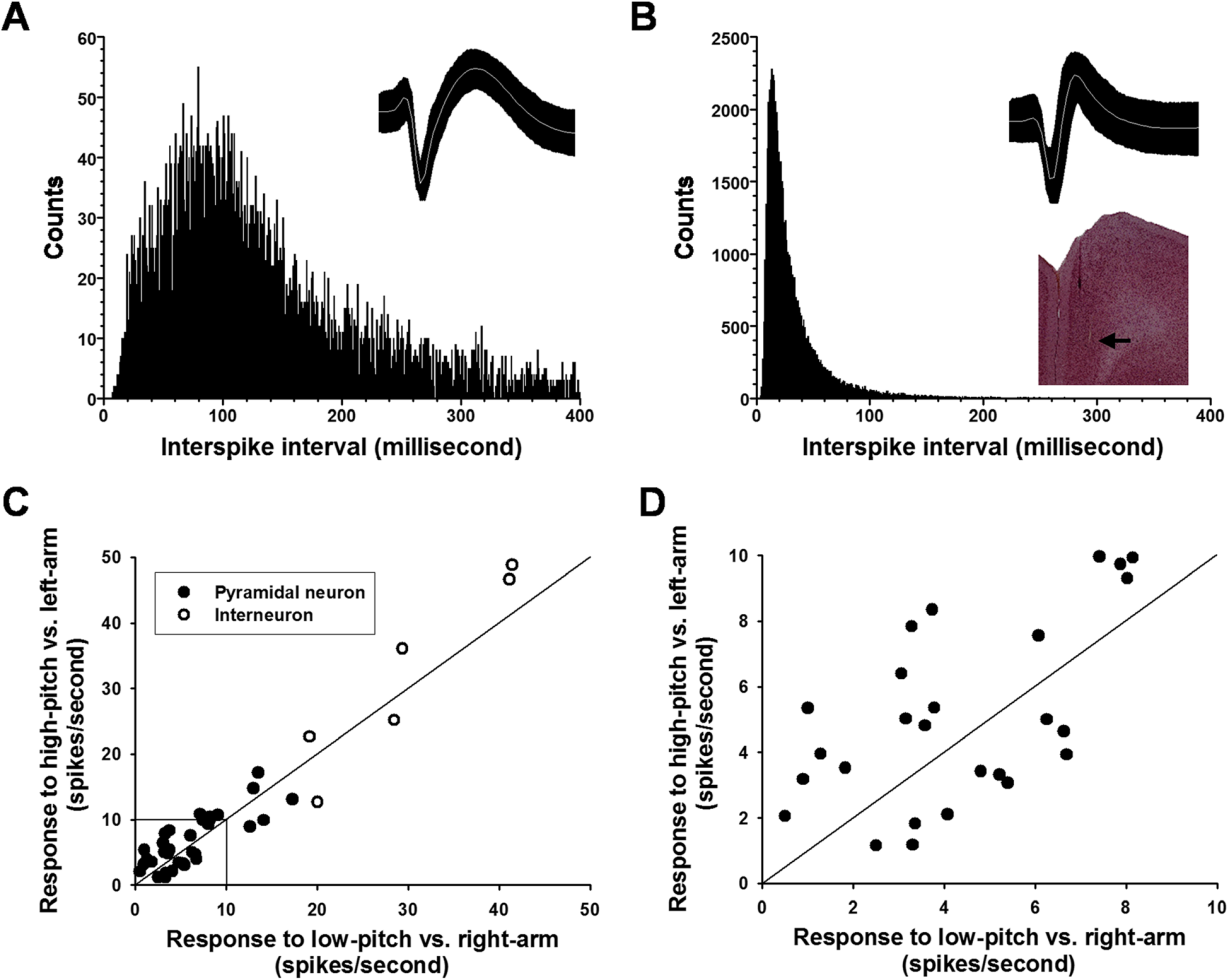
Identification of pyramidal cells versus interneurons and database of preferential cells. **a** Inter-spike interval histogram and average spike waveform of a pyramidal cell. **b** Inter-spike interval histogram and average spike waveform of an interneuron. Pyramidal cells typically fired slowly with wider spike waveform and exhibited longer inter-spike intervals, whereas interneurons typically fired fast with narrow spike waveform and demonstrated shorter inter-spike intervals. Bin width is 1.0 ms. *Insert*: a brain section illustrating the placement of electrode array in the mPFC. **c** Plotting of responses to audio-place associations by preferential cells. The abscissa is the firing response to the high-pitch tone presentation, and the ordinate is that to the low-pitch tone presentation. The cells distributing above the diagonal preferred for the high-pitch vs. left-arm association, and the cells distributing below the diagonal preferred for the low-pitch vs. right-arm association. **d** The enlargement of the squared area in Figure C. Filled circles: pyramidal cells; Open circle: interneurons

A total of 11 rats were used for electrophysiological recordings, and 275 units were recorded and isolated from the mPFC. The units were classified into two categories: pyramidal cell (*n* = 259) and interneuron (*n* = 16), based on their firing rates and waveform characteristics [10]. In general, pyramidal cell has low firing rate and wide waveform (Fig. 3a), whereas interneuron exhibits high firing rate and narrow waveform (Fig. 3b). Of the 275 units, 40 fired differentially during the presentation of the auditory tones (34 pyramidal cells and 6 interneurons; Fig. 3c and d). As shown in Fig. 3c, 25 units demonstrated firing with preference for the ‘high-pitch vs. left-arm’ association, and the remaining 15 units with preference for the ‘low-pitch vs. right-arm’ association.

**Fig. 4:**
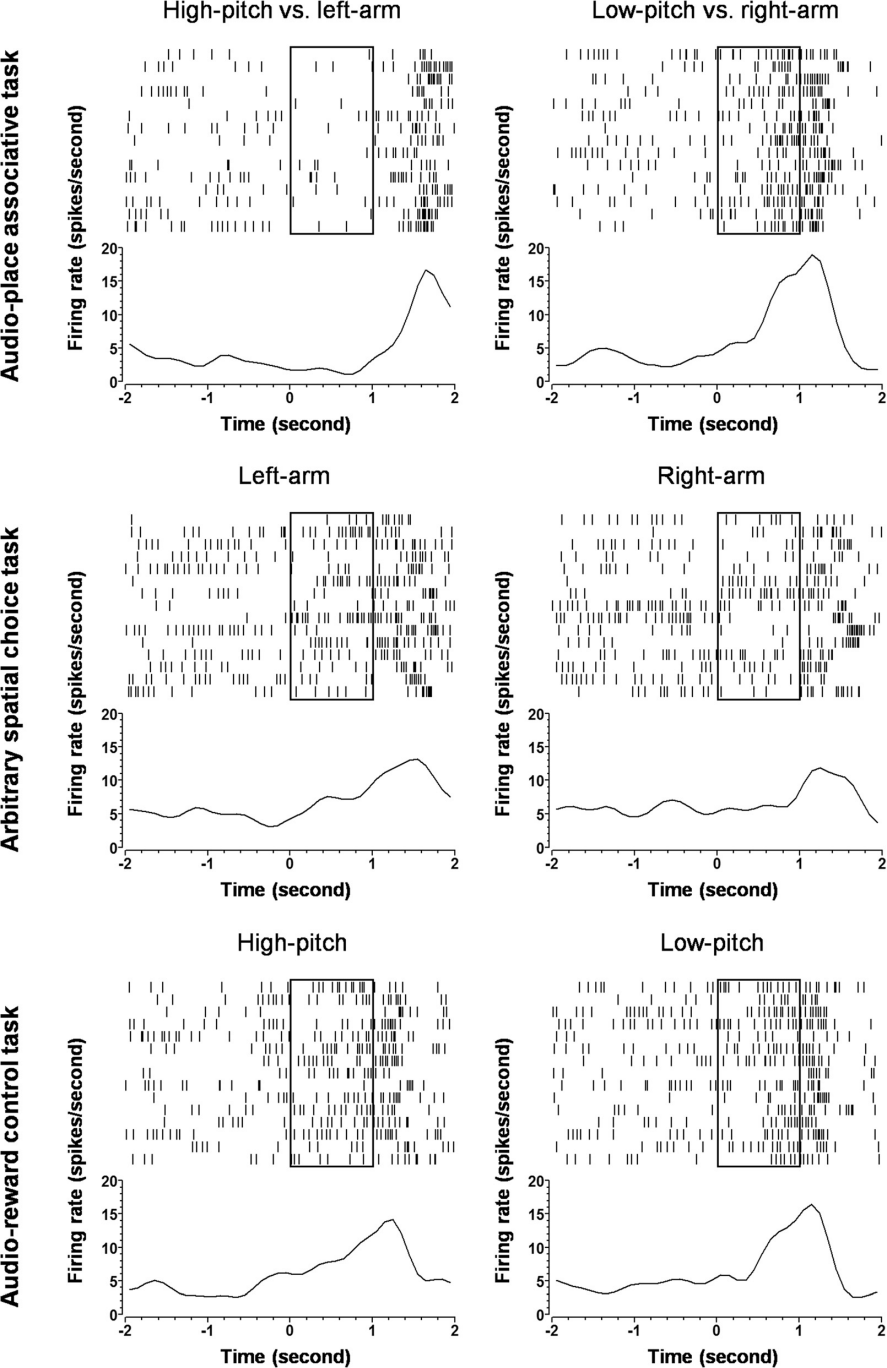
Representative illustrations of the spiking activities of a preferential pyramidal cell. This cell exhibited a preferential firing in response to the low-pitch vs. right-arm association (*upper*), but had no preference for the going-right performance in the arbitrary spatial choice task (*middle*), nor for the low-pitch tone in the audio-reward control task (*lower*). Raster displays show spiking activities in individual trials. Histograms show average spiking activities, smoothed with Gaussian filter. Bin width is 100 ms. The frames in the histograms represent the tone presentation in the audio-place associative task and the audio-reward control task, or the ‘silence’ period in the arbitrary spatial choice task

Figure 4 shows an example of pyramidal cell with preference for the ‘low-pitch vs. right-arm’ association. This unit demonstrated higher firing during the low-pitch presentation than during the high-pitch presentation (*upper*). This firing could not be explained as a response to the low pitch *per se*, as such differential firing did not exist in the audio-reward control task, where the same auditory cues were presented (*lower*), nor as a movement-related activity, since the differential firing was not seen in the arbitrary spatial choice task, where the same ‘going-right’ movement was conducted (*middle*). Thus, it was most likely that the differential firing was encoding the ‘low-pitch vs. right-arm’ association.

**Fig. 5:**
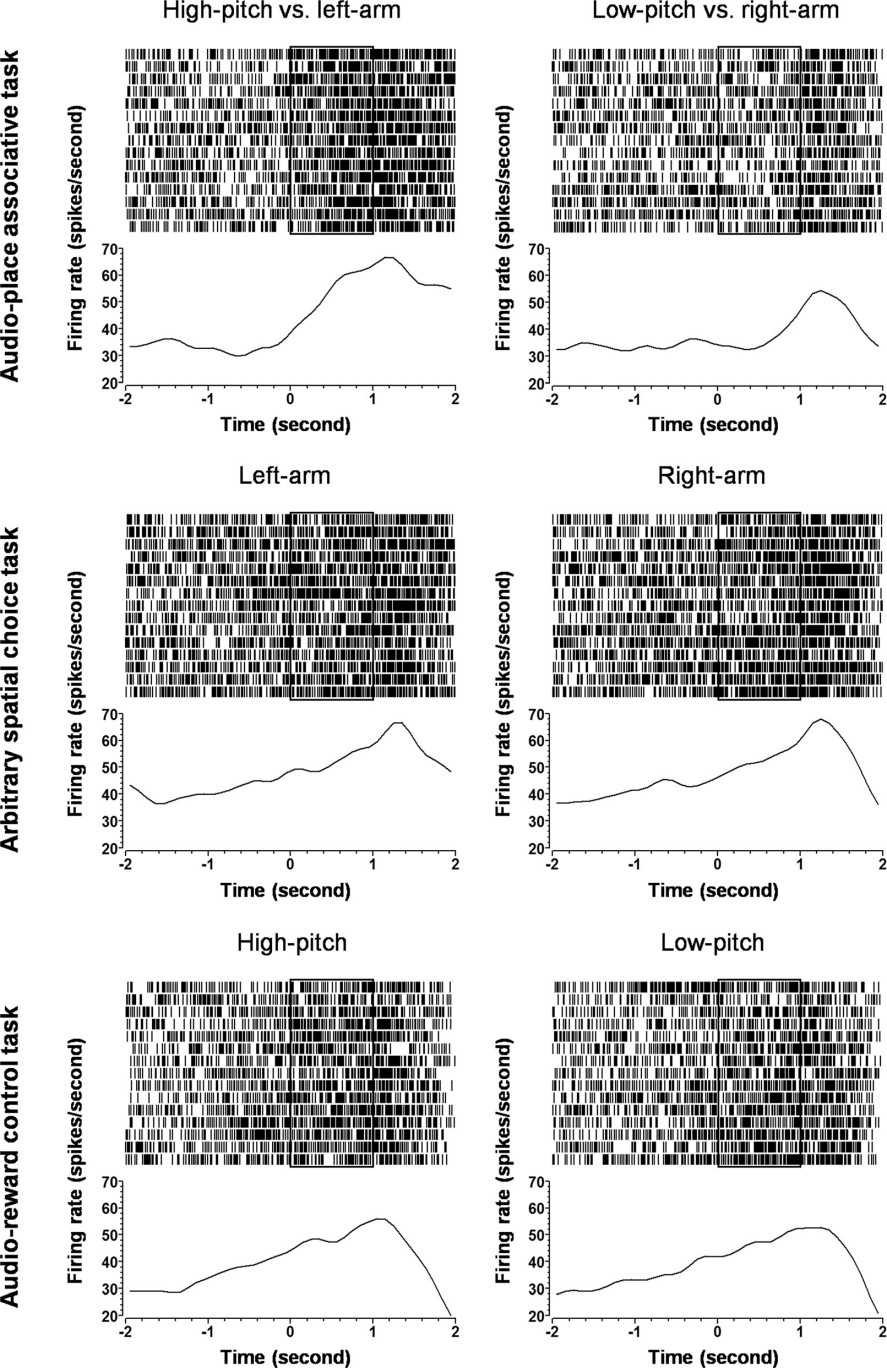
Representative illustrations of the spiking activities of a preferential interneuron. This cell exhibited a preferential firing in response to the ‘high-pitch vs. left-arm’ association (*upper*), but had no preference for the going-left performance in the arbitrary spatial choice task (*middle*), nor for the high-pitch tone in the audio-reward control task (*lower*). Raster displays show spiking activities in individual trials. The configurations of the histograms are the same as in Fig. 4

Figure 5 shows an example of interneuron with preference for the ‘high-pitch vs. left-arm’ association. This unit fired differentially in the audio-place associative task, with a higher firing during the high-pitch presentation than during the low-pitch presentation (*upper*). Such differential firing was not seen in the audio-reward control task (*lower*), nor in the arbitrary spatial choice task (*middle*). Thus, the differential firing was representing the ‘high-pitch vs. left-arm’ association.

### Sequential appearance of firing preference

**Fig. 6:**
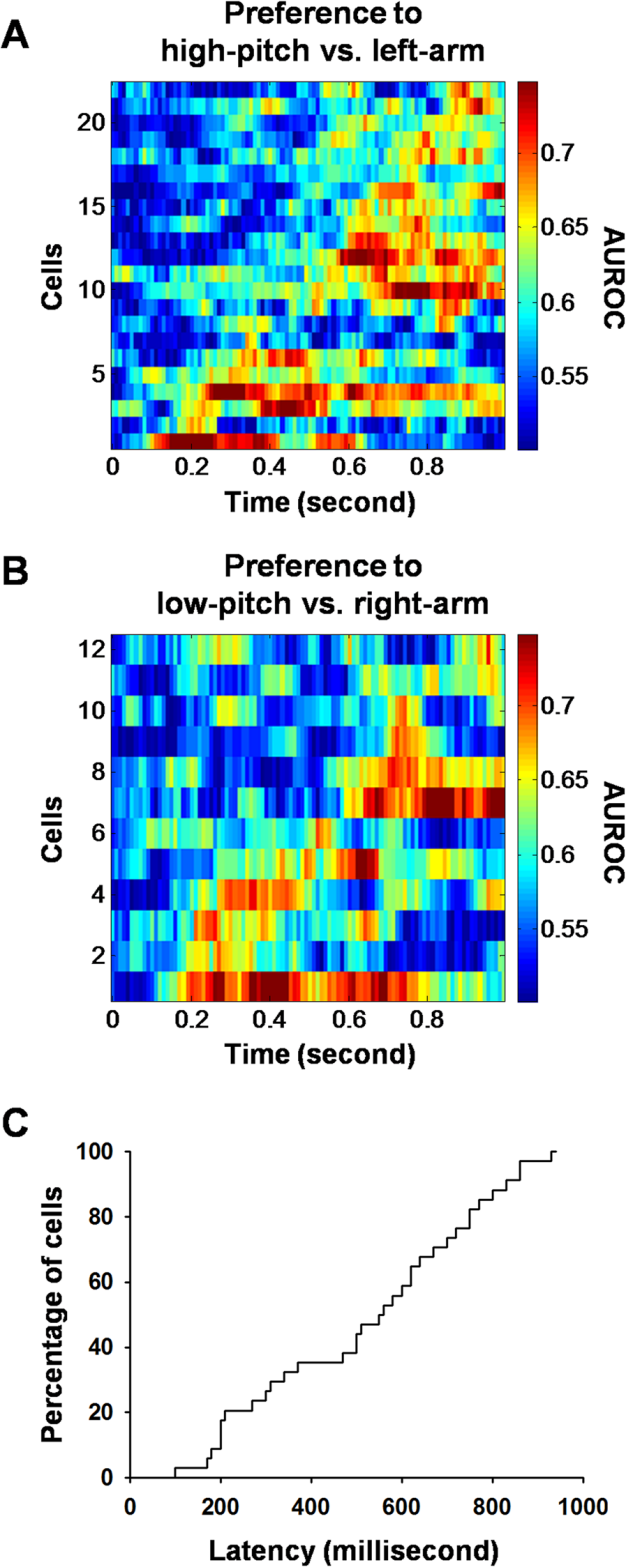
Sequential activities during the tone presentation of cells with preference for the audio-place associations. **a** Cells with preference for the high-pitch vs. left-arm association. **b** Cells with preference for the low-pitch vs. right-arm association. Each row represents the activities of an individual cell. **c** Cumulative distribution of response latencies for all of the preferential cells. Bin width is 10 ms

To examine the temporal sequence of firing preference for the audio-place associations, we performed ROC analysis and calculated the AUROC of the cells with preference for the ‘high-pitch vs. left-arm’ association and those with preference for the ‘low-pitch vs. right-arm’ association. As shown in Fig. 6a and b, the cells with preference for the ‘high-pitch vs. left-arm’ association (*n* = 22) demonstrated sequential appearance of differential firing, so did the cells with preference for the ‘low-pitch vs. right-arm’ association (*n* = 12). Figure 6c shows the cumulative distribution of the appearance latencies for the 34 cells.

### Preference indices in correct vs. incorrect trials

**Fig. 7:**
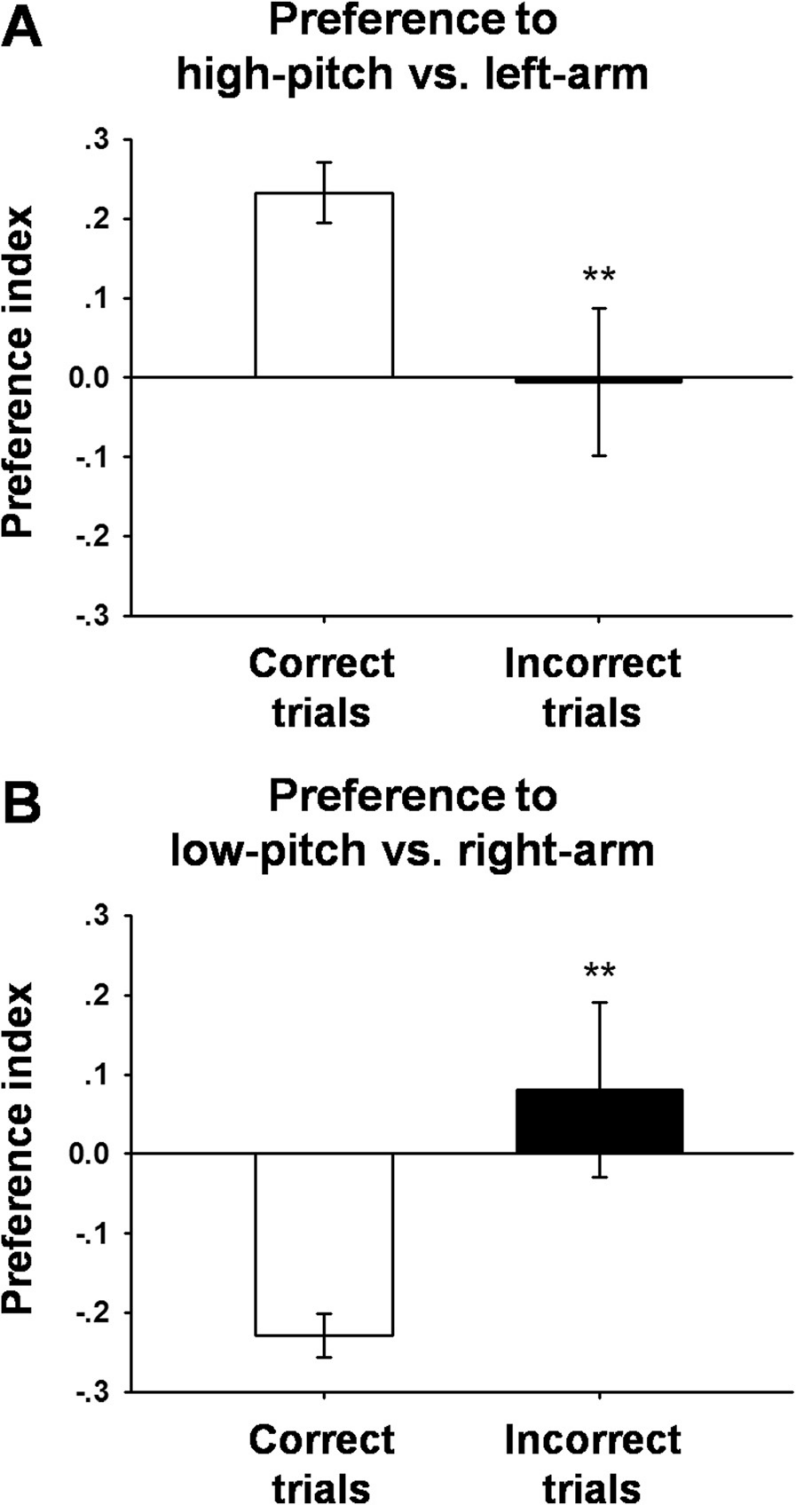
Preference indices of cells in correct and incorrect trials. **a** Cells with preference for the high-pitch vs. left-arm association had preference indices of 0.233 ± 0.038 in correct trials vs. -0.006 ± 0.093 in error trials. **b** Cells with preference for the low-pitch vs. right-arm association had −0.229 ± 0.027 in correct trials vs. 0.081 ± 0.110 in error trials. ** *P* < 0.01, rank-sum test

Preference index reflects the preference strength for the audio-place associations. We compared the preference indices of the preferential cells in correct vs. error trials. As shown in Fig. 7a, the cells with preference for the ‘high-pitch vs. left-arm’ association had a preference index of 0.233 ± 0.038 in correct trials vs. -0.006 ± 0.093 in error trials (*p* < 0.01, rank-sum test, *n* = 25). Similarly, the cells with preference for the ‘low-pitch vs. right-arm’ association had a preference index of −0.229 ± 0.027 in correct trials vs. 0.081 ± 0.110 in error trials (*p* < 0.01, rank-sum test, *n* = 15). This result strongly suggests that the preferential cells were encoding the established associations between the two auditory cues and the two maze arms.

## Discussion

The present study shows that neurons in the mPFC are involved in encoding audio-place associations, supporting the function of mPFC in binding external stimuli with spatial locations [9, 11]. Previous study showed that the medial prefrontal cortex (mPFC) is crucial for object-in-place associational discrimination [9]. For instance, lesions to the mPFC by local infusion of NMDA severely impaired the performance of rats on an object-in-place task. While control rats spent significantly more time exploring a pair of objects that were in different locations compared with the other pair of objects that were in the same locations, rats with mPFC lesion spent almost equal time exploring the two pairs of objects, indicating mPFC involvement in stimulus-place binding [9].

### Importance of the mPFC for audio-place associations

Muscimol inactivation has been widely used to explore functional importance of cortical or subcortical structures [12, 13]. In the present study, reversible inactivation of the mPFC by muscimol severely impaired the performance of rats on the audio-place association task. Analysis of errors revealed that, the win-stay and change-shift strategies were impaired upon intra-mPFC infusion of muscimol, suggesting that the ability to implement the associative rules was affected. This result is well consistent with previous studies showing the essential role of the mPFC in object-place associative tasks [9, 11].

However, because of the non-specific effects of muscimol, it was also possible that muscimol affected the task performance via effects on other functional aspects of the cortex. Indeed, we found that the lose-shift performance was impaired upon muscimol inactivation of the mPFC. The animals showed a tendency to repeat previously-emitted errors, suggesting a deficit in error-correcting ability. This result is well consistent with previous studies in both monkeys and rats, where inactivation of the dlPFC and the mPFC destroyed the error-correcting ability in performance of behavioral tasks [6, 14].

### Representation by mPFC cells of audio-place associations

The ability to integrate information about sensory stimuli and spatial locations is vital for performing the audio-place associative task. Sensory and spatial information are initially processed in largely separate sub-regions of the brain. The mPFC is probably a part of neural networks representing stimulus-place associations. The present study successfully identified cells in the mPFC which encoded specific combinations of the auditory cues and the side arms of the Y-shaped maze. We found that not only pyramidal cells but also interneurons were involved in encoding audio-place associations, although the number of the association-related interneurons was much smaller than that of the pyramidal cells. This was because that the diameter of the electrodes was relative large and it was less possible to encounter interneurons than pyramidal cells. However, it is reasonable to speculate that, both pyramidal cells and interneurons are equally important for encoding audio-place associations, because brain function execution requires local circuits formed by pyramidal cells and interneurons.

The representation of the audio-place associations by the preferential cells could be dissociated from that of the motor performances (running movements) or the physical characteristics of the auditory tones, because there was no differential firing for these cells during the arbitrary spatial choice task performance, where the same motor performances were required, nor during the audio-reward control task performance, where the same tones were presented.

Comparison between the preference indices of the mPFC cells in correct and error trials further revealed the representation of the audio-place associations by the preferential cells. The preference index in error trials was significantly different from that in correct trials. In other words, once a cell did not properly show the association-representing activity, an incorrect response was to be emitted.

We failed to encounter cells with sustained differential firing during the tone-presentation. Most of the cells showed a transient differential discharge. ROC analysis revealed that, the differential discharge appeared sequentially, suggesting that the mPFC cells may carry on audio-place associational information sequentially for information flow among cells.

Mulder et al. reported that mPFC neurons are involved in encoding visuo-reinforcer associations [15]. One may argue that, the preferential cells in the present study were encoding audio-reward instead of audio-place associations. This was not the case, as these cells demonstrated no differential firing in the audio-reward control task.

In summary, the present study provides the evidence that, 1) the mPFC is essential for rats to perform the audio-place associative task, and 2) mPFC cells represent the associations between the auditory stimuli and spatial locations in the task. For future study, the local and trans-cortical circuits underlying audio-place mapping should be addressed, using multi-channel recording technique and optogenetic approaches.

## Materials and methods

### Subjects

Male Sprague–Dawley rats (200–250 g) were purchased from the Shanghai Laboratory Animal Center, Chinese Academy of Science, China. They were housed one per cage under constant temperature (23 ± 1 °C) with a 12/12-h light/dark cycle. Food and water was available *ad libitum*. Surgery was executed after habituation of 7 days to the laboratory vivarium. All experimental protocols were in compliance with the Guide for the Care and Use of Laboratory Animals issued by National Institutes of Health (USA, 1996), and were approved and monitored by the Ethical Committee of Animal Experiments at the Institute of Neurobiology, Fudan University (Shanghai, China).

### Apparatus

Behavioral training was conducted in an automated Y-maze in a room which was moderately illuminated and was rich in extra-maze visual cues. The Y-maze was made up of opaque plastic board, and consisted of one start box (length: 70 cm; width: 15 cm; height: 20 cm) and two side arms (length: 40 cm; width: 15 cm; height: 20 cm). The maze was placed 70 cm above the floor. The start box and side arms each had a retractable door at the entrance. In addition, there was a nozzle at the terminal of each side arm for delivery of water as reward. Infrared light emitting diodes (LEDs) were installed inside the maze and used for detecting the location of rats and recording the time stamp of behavioral events. The maze apparatus was automatically controlled and monitored by a personal computer.

### Behavioral tasks

#### Audio-place associative task

The audio-place associative task required the rats to associate two tones (8 or 1 kHz; 1000 ms) with two location choices, namely, entering the left arm when the high pitch (8 kHz tone) was presented, or entering the right arm when the low pitch (1 kHz tone) was given (Fig. 1a).

To start a trial, a rat was placed in the start box. The rat came to the front of the start-box door and one of the two tones was presented. Upon the termination of the auditory cue, the start-box door was opened, and the rat chose to enter one of two side arms. If the animal entered a correct side arm, it could get water reward (50 μL) at the terminal of the arm. And if the animal entered an incorrect side arm, no water reward was delivered and a feedback auditory signal (300 Hz; 500 ms) was presented. Then, the animal returned to the start box and the start-box door was closed immediately. The two auditory cues were presented in a semi-randomized order.

#### Arbitrary spatial choice task

Arbitrary spatial choice task was modified from the audio-place associative task, by replacing the auditory cues with a period of silence (1000 ms), that is, no tone was presented. To perform the task, rats were required to visit the two side arms arbitrarily, without the guidance of auditory cues. This task was used as a control task to test whether mPFC cells would show a response to movement-related factors.

#### Audio-reward control task

The apparatus for the audio-reward control task was modified from the Y-maze, by blocking the entrance of the two side arms, and resetting a water-delivery nozzle at the intersection of the side arms (Fig. 1b). To start a trial, a rat was placed in the start box. The rat came to the front of the start-box door, and one of the two tones, as used in the audio-place associative task, was presented. Upon the termination of the auditory cue, the start-box door was opened, and the rat went straightway to the intersection, where water reward (50 μL) was delivered regardless of the high- or low-pitch had been presented. This task was used as a control task to test if mPFC cells would show a differential response to physical characteristics of the auditory tones.

### Training procedure

Before training, rats were handled by the experimenter 10 min per day for one week. Then, the rats were fully deprived of water for two days in order to induce motivation for task performance. Later, the rats were acclimated to the Y-maze and were trained to perform the audio-place associative task. Each daily session consisted of 150 trials. An error-correction procedure was introduced, that is, if the rats made an incorrect choice, the same auditory cue was presented again to give the rats a chance to adjust behavioral choice. Performance with a correct rate of >80 % in two consecutive daily sessions was considered as the criteria for learning.

Usually, it took 2–3 weeks for rats to learn the task. On training days, the rats were awarded with water during training, and given additional water after training, so that each animal could get a total of 30 mL water each day. For example, if a rat was reward 20 mL water during training, it was given additional 10 mL water after the training session.

After the rats learned the audio-place associative task, one group of them were used for behavioral experiments with mPFC inactivation, and the other group were further trained on the arbitrary spatial choice task and the audio-reward control task, and thereafter used for electrophysiological experiments (Fig. 1c).

### Surgery

#### Implantation of guide cannula for drug administration

Rats were anesthetized with sodium pentobarbital (40 mg/kg, i.p.) and restrained in a stereotaxic frame (Narishige SN-2, Japan). Then, the rats were bilaterally implanted with stainless steel guide cannula (23 gauge, 8 mm length). The tip of the guide cannula was placed at 3.5 mm anterior to bregma, 0.5 mm lateral to midline suture, and 2 mm ventral to skull surface. These stereotaxic coordinates were based on the Paxinos and Watson [16]. The guide cannulas were anchored to the skull with stainless steel screws and dental cement. A stylus was inserted into the guide cannulas to prevent clogging and reduce the risk of infection. Rats were allowed to recover for 7 days with free access to food and water before behavioral experiments.

#### Implantation of microelectrode array for spike recording

Microelectrode array was consisted of 16 microelectrodes (Formvar-insulated nichrome wires, 35 μm in diameter) in a 2 × 8 configuration with 200 μm between electrodes, and was drivable by turning the screw of microelectrode array. Immediately prior to implantation, these electrode wires were freshly cut with surgical scissor to reduce the impedance to 0.5 ~ 1 MΩ (measured at 500 Hz).

For implantation of microelectrode array, rats were anesthetized with sodium pentobarbital (40 mg/kg, i.p.) and mounted on the stereotaxic frame. Microelectrode array was implanted in the left mPFC (2.5 ~ 4.5 mm anterior to bregma, 0.3 ~ 0.8 mm lateral to midline suture, and 2.0 mm ventral to brain surface). The array was anchored to the skull with stainless steel screws and dental cement. A stylus was inserted into the connector of the array to prevent clogging. Rats were allowed to recover for 7 days with free access to food and water before behavioral training. For recordings of spike activities, the array was lowered in steps of 80 μm every session throughout the recording experiments.

### Drug administration

In order to assess the importance of the mPFC for the audio-place associative task performance, we locally infused the GABA_A_ receptor agonist muscimol (Sigma, Missouri, USA) into the mPFC to reversibly inactivate this cortical region. For muscimol infusion, rats were gently held. The stylus was removed from the guide cannula, and an injection needle (30 gauge, 10 mm length) was inserted into the guide cannula. The tip of the injection needle was 2 mm lower than that of the guide cannula. Muscimol solution (1 μg/μL) or saline was bilaterally infused into the mPFC (0.5 μl each side) at a rate of 0.25 μl/min. Bilateral infusions were performed simultaneously. The injection needle remained in the guide cannula for additional 3 min after the infusion was completed. Behavioral test was conducted 15 min later.

### Recording of neuronal discharge

After recovery from the surgery for implantation of microelectrode array, rats received training on the audio-place associative task, arbitrary spatial choice task, and audio-reward control task sequentially. Neuronal activities were recorded using neural signal acquiring system (Cyberkinetics, USA). Unit signals were amplified 5000×, band-pass filtered between 500 Hz and 7.5 kHz and digitized at 30 kHz. The time stamp of the task events were integrated with the spike data on line. Raw data were stored in a computer for off-line analysis. The microelectrode array was advanced in an 80-μm step after a daily recording session, until the tips of the electrodes reached the depth of 4.0 mm below the cortical surface.

### Data analysis

#### mPFC inactivation experiment

Rats usually made three types of error when performing the audio-place associative task: they repeated incorrect choice on repeat trial (*Lose-shift failure*), or they did not repeat correct choice on repeat trial (*Win-stay failure*), where the auditory cue was the same as in the previous trial, or they did not change choice on change trial, where the auditory cue was changed from the previous trial (*Change-shift failure*). Correct rate of performance (percentage of correct trials), percentage of error, and reaction time were statistically compared between the muscimol and saline groups, using Student’s *t*-test. Significant level was set at 0.05. Data in the text and figures are expressed as means ± SEM (standard error of mean).

#### Electrophysiological recording

Single units were isolated offline with the offline sorter (Plexon Inc., Dallas, TX) using principal components analysis and a template-matching algorithm. Waveforms with inter-spike interval of <2.0 ms were excluded. Cells were selected for analysis if they reached the following criteria: 1) signal-to-noise ratio was larger than 3:1; 2) spiking activity was stable during a whole recording session; and 3) inter-trial baseline firing was stable during performance of the three tasks. The period of cue presentation was the vital time window for the rats to execute audio-place associations. Thus, electrophysiological analysis focused particularly on this time window.

A cell was taken as a preferential cell if it showed a change in firing in the audio-place associative task but not in the arbitrary spatial choice task and the audio-reward control task. Statistical significance (*p* < 0.05) was assessed using rank-sum test. All statistical analysis was conducted using Matlab software (Mathworks, USA).

### ROC analysis

To characterize the time course of preference for the audio-place associations, we compared firing activity during the cue presentation, using the sliding receiver operating characteristic (ROC) analysis [17]. The ROC analysis was performed over the window of the tone presentation (100 ms bin, 10 ms step). The area under the ROC curve (AUROC) was taken as a quantitative measure for testing how well a cell preferred for the audio-place associations.

While AUROC of 0.5 indicated no preference, AUROC of 1.0 represented a perfect preference for the audio-place associations, with AUROC of 0.65 as the threshold of preference. The criterion value (0.65) was determined by calculating the 99^th^ percentile of AUROC during the baseline period. The 2-s period before cue presentation was used as a baseline period. Preference latency was defined as the time where AUROC exceed 0.65 for the first time in three consecutive sliding bins.

### Preference index

The preference index was computed to quantify the preference strength for audio-place associations across population of cells in the audio-place associative task. Preference index (PI) was calculated using the following formula:

$$ PI=\left( Activit{y}_{high- pitch}- Activit{y}_{low- pitch}\right)/\left( Activit{y}_{high- pitch}+ Activit{y}_{low- pitch}\right) $$

Where, Activity_high-pitch_ was the firing activity in response to the high pitch, and Activity_low-pitch_ was the firing activity to the low pitch in the task. The PI ranged from +1.0 (complete preference for the ‘high-pitch vs. left-arm’ association) to −1.0 (complete preference for the ‘low-pitch vs. right-arm’ association). PI of 0 indicated no preference for the audio-place associations.

### Histology

Rats were anesthetized with an overdose of sodium pentobarbital (80 mg/kg, i.p.) and transcardially perfused with 0.9 % saline solution, followed by 10 % formalin. After decapitation, the brains were removed and submerged into sucrose solutions ranging from 10, 20 to 30 % concentration until sank to the bottom. Then, the brains were cut into 40 μm sections with a freezing microtome (Leica, Germany). The brain sections were mounted on gelatin-coated glass slides and stained with neutral red for histological examination.
